# The Liver Left Behind: GLP-1 Receptor Agonists and the Prospect of Metabolic Adjuvant Therapy After HCC Resection

**DOI:** 10.3390/metabo16070499

**Published:** 2026-07-15

**Authors:** Amedeo Lonardo, Ralf Weiskirchen

**Affiliations:** 1 Independent Researcher, 41100 Modena, Italy; 2 Institute of Molecular Pathobiochemistry, Experimental Gene Therapy and Clinical Chemistry (IFMPEGKC), RWTH University Hospital Aachen, D-52074 Aachen, Germany; rweiskirchen@ukaachen.de

Hepatocellular carcinoma (HCC), the most common histological type of primary liver cancer (PLC), remains one of the most consequential malignancies arising in chronic liver disease, in part because its incidence and mortality rates remain closely aligned [1]. Although cirrhosis due to viral hepatitis, alcohol-related liver disease, or other chronic injuries has long provided the dominant clinical framework for HCC risk, this paradigm is being reshaped by the global expansion of metabolic dysfunction-associated steatotic liver disease (MASLD), obesity, and type 2 diabetes (T2D). These conditions enlarge the population at risk and blur traditional surveillance boundaries, as HCC may develop not only in advanced fibrosis or cirrhosis, but also, in selected patients, in non-cirrhotic metabolic liver disease [2,3].

In 2021, metabolic risk factors contributed substantially to the global burden of PLC: high body mass index (BMI) and/or high fasting plasma glucose accounted for an estimated 59,970 deaths [4]. Cases of PLC attributable to metabolic risk factors have increased steadily from 1990 to 2021, with the burdens of obesity and dysglycemia being higher in men than in women [4].

The biological distinctiveness of metabolic HCC is increasingly recognized. In MASLD, sustained nutrient excess, insulin resistance, and lipotoxic stress converge to promote hepatocellular injury, inflammatory signaling, fibrogenesis, and oxidative DNA damage. In parallel, malignant transformation is supported by metabolic reprogramming, including altered lipid handling and enhanced anabolic flux, as well as by remodeling the tumor microenvironment toward immune dysfunction [5]. Exhausted lymphocyte populations, tumor-associated macrophages, and other immunosuppressive cellular networks may facilitate tumor progression and may also influence sensitivity to immune checkpoint blockade [5]. Thus, metabolic HCC should not be regarded simply as HCC occurring in patients with metabolic comorbidity, but rather as a disease shaped by sustained crosstalk among systemic metabolic dysfunction, the chronically injured liver, and the evolving tumor ecosystem [5,6,7].

A central clinical challenge is to define which individuals with T2D warrant intensified HCC surveillance. The absolute incidence of HCC in diabetes remains modest when considered against the very large and growing denominator of affected individuals, making indiscriminate screening impractical and potentially inefficient [8]. Conversely, failure to identify high-risk subgroups may delay diagnosis in patients who fall outside conventional cirrhosis-based surveillance pathways. Scalable risk-stratification approaches are therefore required, integrating routine clinical variables, non-invasive fibrosis assessment, metabolic severity, liver-related biomarkers, and, ultimately, molecular or imaging signatures of hepatocarcinogenic risk [8]. Such tools will be essential not only for surveillance, but also for prevention trials evaluating whether antidiabetic agents—including sodium–glucose cotransporter 2 inhibitors and incretin-based therapies—can potentially modify the trajectory from metabolic dysfunction-associated steatohepatitis (MASH) to HCC [8].

The implications of T2D and MASLD extend beyond cancer incidence to encompass tumor biology, treatment tolerance, and long-term outcome. In patients with established HCC, coexisting T2D has been associated with poorer prognosis, potentially reflecting a combination of adverse tumor features, including microvascular invasion, and the persistent inflammatory and metabolic perturbations of the surrounding liver [9,10]. This hepatic field effect is particularly relevant after curative-intent resection or ablation, when recurrence may arise from residual microscopic disease or from de novo tumor formation within a liver that remains biologically permissive. Although effective adjuvant strategies are urgently needed, clinical trials have not consistently shown durable benefit, and contemporary AASLD and EASL guidelines therefore remain cautious and do not recommend routine postoperative systemic therapy after curative-intent treatment [11,12,13,14,15].

Diabetes management should be regarded as an integral component of long-term care after liver resection for HCC, rather than as a parallel metabolic concern. The available evidence indicates that diabetes is not merely a background comorbidity: impaired glycemic control is associated with recurrence risk after curative-intent hepatectomy [16], and the coexistence of obesity and diabetes appears to amplify late recurrence and worsen overall prognosis [17]. These observations are biologically plausible, as insulin resistance, chronic inflammation, steatotic liver disease, and immune–metabolic dysfunction may all create a pro-tumorigenic hepatic microenvironment during postoperative regeneration and immune surveillance. Importantly, the signal is not reduced to the use of any single antidiabetic agent; in diabetic patients undergoing initial resection, poor glycemic control, rather than metformin exposure alone, emerges as the clinically relevant determinant [16]. Thus, postoperative HCC care should incorporate structured diabetes assessment, individualized HbA1c targets, nutritional and weight-management strategies, optimization of liver function, and coordinated follow-up among hepatobiliary surgeons, hepatologists, and diabetologists. Embedding metabolic control within lifelong oncological surveillance could refine risk stratification, guide follow-up intensity, and potentially reduce late recurrence, thereby transforming diabetes management into a modifiable pillar of survivorship after HCC surgery [16,17].

Together, these observations support a broader prevention paradigm for metabolic HCC. Durable recurrence reduction is unlikely to follow from residual tumor control alone; it will also require strategies that reshape the injured, insulin-resistant and immunologically remodeled hepatic milieu that supports de novo carcinogenesis. The emerging priority is therefore to integrate oncological control with metabolic and liver-directed prevention, aligning surveillance, systemic risk modification and adjuvant innovation with the biology of the host organ [18].

Against this background, Xiang and colleagues address a timely clinical question: in patients with HCC and T2D undergoing R0 resection, is postoperative initiation of a glucagon-like peptide-1 receptor agonist (GLP-1RA), rather than a dipeptidyl peptidase-4 inhibitor (DPP-4i), associated with improved long-term outcomes?

Their nationwide, multicenter target-trial emulation, based on electronic medical records from 36 hospitals in China, provides a cautiously affirmative signal. Among adults with HCC and T2D after curative-intent resection, GLP-1RA initiation was associated with longer recurrence-free and overall survival than DPP-4i initiation. In the weighted intention-to-treat analysis, the association was modest but clinically meaningful for recurrence-free survival and more pronounced for overall survival, with hazard ratios of 0.80 and 0.58, respectively.

The findings reopen a broader question: can postoperative metabolic therapy become part of the HCC adjuvant landscape, not as conventional tumor-directed treatment, but as modification of the host and hepatic substrate in which recurrence develops? This question is timely, as adjuvant sorafenib failed to improve outcomes in STORM [11], and longer follow-up from IMbrave050 has questioned the durability of the initial recurrence-free survival benefit with atezolizumab plus bevacizumab [13]. Thus, despite curative-intent surgery, recurrence remains common and the systemic adjuvant gap persists.

The conceptual strength of the study is its framing. Xiang and colleagues do not position GLP-1RAs as another anticancer drug; rather, they test whether GLP-1RA therapy may alter the background biology that sustains carcinogenesis in the remnant liver. The hypothesis is plausible: beyond glucose lowering, GLP-1RAs promote weight loss and confer cardiovascular and kidney protection, and experimental and clinical data link GLP-1 signaling to improvements in steatosis, insulin resistance, inflammation and fibrotic pathways. Observational studies also associate GLP-1RA use with fewer major liver-related outcomes and less hepatic decompensation in T2D. The novelty here is the extension of this substrate-focused hypothesis to postoperative HCC, where recurrence-free and overall survival are clinically decisive outcomes.

Design merits attention. The authors used an active-comparator, new-user target-trial emulation, rather than a simple treated-versus-untreated comparison. Eligible patients had histologically confirmed HCC and T2D, underwent R0 resection, and initiated either a GLP-1RA or DPP-4i within 90 postoperative days. Time zero was the first qualifying postoperative prescription or order, reducing immortal-time and time-alignment bias. The primary estimand was the intention-to-treat effect; a prespecified per-protocol analysis assessed sustained adherence, censoring at crossover or permanent discontinuation.

The comparator is also clinically apt. DPP-4 inhibitors share indications with GLP-1RAs in T2D but lack many of their extra-glycemic effects. Comparing new users of these two drug classes therefore approximates a realistic postoperative treatment choice, while avoiding the broader confounding inherent in treated-versus-untreated contrasts.

The cohort selection illustrates both the scale and specificity. Of 42,855 patients with HCC and T2D who underwent liver resection, 5973 had a qualifying postoperative GLP-1RA or DPP-4i prescription. After exclusions, the analytical cohort included 1249 patients—723 DPP-4i initiators and 526 GLP-1RA initiators—and median follow-up was 50.8 months, allowing for assessment of clinically relevant recurrence and survival outcomes.

As expected, baseline imbalances were present before adjustment: GLP-1RA initiators were treated more often in recent years, more commonly had MASH and cirrhosis, and differed in liver-function profiles. Stabilized inverse-probability weighting achieved covariate balance, with all standardized mean differences below 0.10 and an effective sample size of 1066 in the primary intention-to-treat analysis.

The statistical approach was appropriate for the endpoints. Death without prior recurrence was treated as a competing event, with cause-specific Cox models for recurrence. Overall survival was analyzed using weighted Kaplan–Meier and Cox models, with center-stratified models and robust standard errors accounting for the multicenter structure.

The primary results are clinically notable. During follow-up, 606 recurrences and 447 deaths occurred, and weighted median recurrence-free survival was 62.6 months with GLP-1RA initiation and 42.1 months with DPP-4i initiation, corresponding to a cause-specific hazard ratio of 0.80 (95% CI, 0.67–0.96; *p* = 0.016). Adjusted recurrence risk differences favored GLP-1RA initiation by 6.1 percentage points at 36 months and 7.7 percentage points at 60 months. Overall survival also favored GLP-1RA initiation: median survival was not reached versus 57.8 months with DPP-4i, with a weighted hazard ratio of 0.58 (95% CI, 0.47–0.71; *p* < 0.001).

Sensitivity analyses strengthened but did not prove the signal. E-values were 1.81 for recurrence-free survival and 2.84 for overall survival, indicating the magnitude of unmeasured confounding needed to explain away the associations. Findings were directionally consistent across alternative weight truncation, overlap weighting, propensity-score matching and a 3-month landmark analysis. Restricted mean survival time contrasts also favored GLP-1RA initiation for recurrence-free survival at 36 months and overall survival at 60 months.

Outcome controls add further reassurance. GLP-1RA initiation was strongly associated with >5% weight loss at 12 months versus DPP-4i initiation (risk ratio, 3.77), consistent with an expected pharmacological effect. By contrast, no association was seen with hospitalization for non-cancer trauma or injury, arguing against a broad nonspecific bias pattern.

The timing of the benefit is especially informative. Recurrence-free survival curves separated only after approximately 12 months, a pattern more compatible with delayed modification of the diseased hepatic field than with early suppression of residual tumor burden. Late HCC recurrence is often field-driven; within this model, weight loss, improved insulin resistance, reduced steatosis, attenuated inflammation and possible fibrotic remodeling could plausibly translate into lower recurrence over time.

The analogy with perioperative antiviral therapy for chronic hepatitis B is useful. Such treatment improves long-term outcomes by controlling the hepatic substrate rather than by acting as cytotoxic therapy. If GLP-1RAs reduce recurrence, they may therefore be best viewed as metabolic, rather than conventional oncological, adjuvant therapy.

The stronger association with overall survival (compared with recurrence-free survival) also fits this metabolic interpretation. Overall survival reflects not only recurrence but also post-recurrence outcomes and non-cancer mortality. Because GLP-1RAs improve weight, glycemia and cardiometabolic risk, they may benefit postoperative patients with T2D through several pathways, particularly in those with competing cardiovascular, renal and hepatic risks.

Subgroup analyses are hypothesis-generating. Associations appeared more pronounced in patients with MASH and microvascular invasion and attenuated among current smokers. Heterogeneity was also suggested by BCLC stage and alpha-fetoprotein category. These signals are biologically plausible, because MASH marks a modifiable inflammatory–metabolic substrate and microvascular invasion identifies higher oncological risk. However, several strata contained few events, and the results should not guide practice without prospective confirmation.

Per-protocol analyses were supportive but cautionary. Discontinuation was the dominant adherence-related reason for censoring and was more frequent with GLP-1RAs than with DPP-4 inhibitors. By 36 months, adherence in the recurrence-free survival analysis had fallen to 21.0% for GLP-1RA and 43.0% for DPP-4i. Thus, the feasibility of metabolic adjuvant therapy will depend not only on efficacy but also on tolerability, cost, persistence and postoperative practicality.

Time-specific per-protocol estimates favored GLP-1RA initiation for both endpoints, with larger apparent effects than in the intention-to-treat analysis. Yet proportional hazards assumptions were not met, and the effective sample size was reduced after censoring adjustment. These estimates should therefore be read as consistent with the primary findings, not as definitive measures of sustained-adherence benefit.

Additionally, the study by Xiang et al. [18] does not clarify the biochemical pathomechanisms involved. Emerging metabolomic and lipidomic studies suggest that the metabolic response to GLP-1 receptor agonists (GLP-1RAs) is not simply a downstream consequence of improved glycemia or weight loss, but reflects a coordinated remodeling of lipid handling, mitochondrial substrate use and inflammatory bioactive lipid signaling. In recent-onset T2D, untargeted LC-MS profiling showed that dulaglutide and liraglutide partially reversed the diabetes-associated serum metabolome, with the most consistent treatment-associated signature mapping to glycerophospholipid metabolism; a total of 46 and 45 metabolites, respectively, changed after 12 weeks, and pathway-level integration implicated insulin resistance and T2D pathways, while network inference highlighted GLP1R-related signaling nodes, including GLP-1R, GNAS and GCG [19]. This lipid-centered signature is biologically plausible: phosphatidylcholines, lysophosphatidylcholines and lysophosphatidylethanolamines regulate membrane composition, lipoprotein transport, lipotoxic stress and immune-cell activation, and disturbances in these species have been linked to insulin resistance, incident diabetes, cardiovascular risk and MASLD progression [20,21,22,23]. Exenatide studies extend this concept by showing that GLP-1RA exposure modifies sphingomyelins, ceramides, LPCs, LPEs, phosphatidylethanolamines and phosphatidylcholines in parallel with improvements in HbA1c and lipid parameters, consistent with reduced lipotoxic intermediates and improved cardiometabolic lipid flux [24]. Importantly, metabolomics may also stratify therapeutic heterogeneity: in 93 Chinese adults with T2D, baseline HbA1c and HDL-C combined with lower butenylcarnitine and higher LysoPC(18:2) and PC(20:3/20:3) predicted exenatide glucose-lowering response with an AUC of 0.895 [25]. In obesity, liraglutide 3 mg has likewise been associated with broad plasma metabolomic shifts, including oxylipins, arachidonic-acid derivatives, phosphoglycerophosphates, N-acyl amino acids, steroid hormones and bile acids, supporting an integrated effect on inflammatory lipid mediators, enterohepatic signaling and energy balance [26]. Thus, the metabolomic phenotype of GLP-1RA treatment converges on a reproducible axis of glycerophospholipid and sphingolipid remodeling, attenuation of ceramide/LPC-related risk signals, and restoration of lipid species associated with insulin sensitivity, vascular protection, and probably HCC prevention (Figure 1).

However, the field remains limited by small cohorts, short follow-up, platform-dependent metabolite annotation, and incomplete adjustment for weight loss, diet, concomitant therapies, and renal or hepatic function. Future studies should combine targeted quantitative lipidomics, flux analyses, tissue-resolved metabolomics, and longitudinal clinical endpoints to determine whether these metabolites are causal mediators, pharmacodynamic biomarkers, or predictors for the precision use of GLP-1RAs.

Several caveats temper interpretation. Residual confounding remains possible, particularly for frailty, lifestyle, socioeconomic factors, and clinician judgment. Exposure was inferred from prescriptions rather than confirmed use, and postoperative discontinuation or switching may have reflected tolerability, cost, or clinical deterioration. Recurrence ascertainment also depended on imaging practices and documentation, which may vary across centers and over time. The per-protocol analyses were more model-dependent because they required adjustment for informative censoring, and subgroup findings were exploratory, with some sparse strata. Finally, the cohort was drawn from Chinese centers, with a high prevalence of hepatitis B virus infection, limiting immediate extrapolation to populations dominated by MASLD, alcohol-related liver disease, or hepatitis C.

The study also has important strengths. Its target-trial emulation design aligns eligibility, treatment initiation, and time zero, and compares new users of two active glucose-lowering strategies. The analysis accounts for the competing risk of death, uses multiple approaches to control confounding, reports absolute as well as relative effects, and includes outcome-control and per-protocol analyses. These features cannot substitute for randomization, but they make the observational signal more coherent and clinically interpretable.

The clinical message should therefore remain measured. These data do not establish GLP-1RAs as standard adjuvant therapy after HCC resection, nor do they prove superiority over all other diabetes treatments. They address a narrower, but clinically relevant, decision: initiating a GLP-1RA rather than a DPP-4 inhibitor after surgery. In suitable patients with T2D, obesity, MASH or fibrotic liver disease, possible liver-related and oncological benefits may now enter multidisciplinary discussions, alongside tolerability, contraindications, cost, nutrition, patient preference, and the high discontinuation rates seen in routine care.

The research agenda is clearer: prospective trials should test GLP-1RA-based postoperative strategies in patients with HCC, T2D and metabolic liver disease, with stratification by MASH, fibrosis, viral hepatitis, microvascular invasion, tumor burden, and cardiometabolic risk. They should distinguish early recurrence, more likely to reflect residual tumor biology, from late recurrence, more likely to reflect the diseased hepatic field. Embedded mechanistic studies should assess weight, glycemic control, liver fat, fibrosis, inflammation, decompensation, cardiovascular and renal outcomes, adherence, and patient-reported tolerability. Crucially, trials must determine whether any survival advantage reflects fewer recurrences, better outcomes after recurrence, lower non-cancer mortality, or a combination of these effects.

More broadly, HCC recurrence should not be viewed solely as a failure of tumor eradication. The remnant liver is an active biological terrain shaped by viral injury, metabolic dysfunction, inflammation, fibrosis, and systemic comorbidity. In patients with T2D, this terrain may be modifiable. GLP-1RAs are attractive not because they are proven anticancer drugs, but because they target several upstream drivers of hepatic risk.

Xiang and colleagues therefore provide an important observational signal, not a mandate for practice change. Their findings should encourage closer collaboration among oncologists, hepatologists, surgeons, and endocrinologists, and should prompt prospective trials designed around the metabolic and fibrotic biology of recurrence.

If confirmed, this approach could shift postoperative HCC care from a narrow focus on residual tumor control towards modification of the remnant-liver risk. For now, the study changes the question: in HCC complicated by T2D, diabetes therapy may be more than metabolic housekeeping, and the liver left behind may be a therapeutic target on its own.

## Figures and Tables

**Figure 1:**
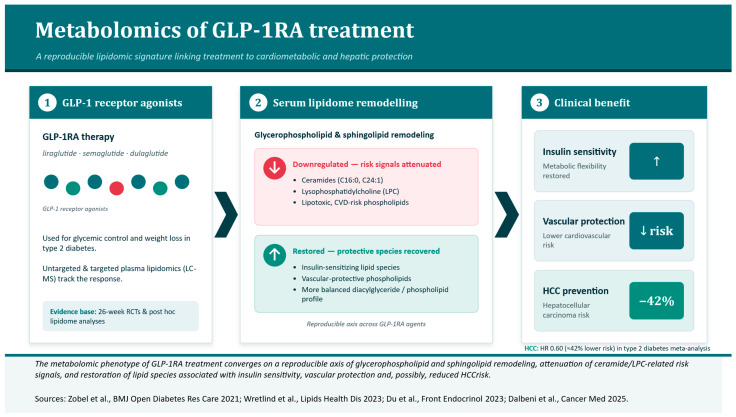
Proposed lipidomic and hepatometabolic framework linking GLP-1 receptor agonist therapy to remnant-liver risk modification after hepatocellular carcinoma treatment. The figure summarizes a putative pathway in which GLP-1 receptor agonists (GLP-1RAs) act upstream of hepatocarcinogenesis by improving adiposity, insulin resistance, hepatic steatosis, inflammatory tone, and lipotoxic lipid signaling rather than by functioning as direct cytotoxic antitumor agents. In patients with type 2 diabetes and metabolic dysfunction-associated steatotic liver disease, these effects may reduce the carcinogenic “field” that persists in the remnant liver after curative-intent resection and may therefore contribute to delayed reductions in de novo or field-driven HCC recurrence. This prevention-oriented interpretation is consistent with other studies, including publications by Dalbeni et al. [27], Zobel et al. [28], Wretlind et al. [29] and Du et al [19]. The proposed framework remains hypothesis-generating: whether these circulating lipidomic changes are causal mediators of HCC prevention, pharmacodynamic biomarkers of GLP-1RA exposure, or correlates of weight loss and improved systemic metabolism requires prospective validation with targeted lipidomics, tissue-resolved liver analyses, flux studies, and longitudinal oncological endpoints.

## Data Availability

No new data were created or analyzed in this study. Data sharing is not applicable to this article.

## Abbreviations

The following abbreviations are used in this manuscript:

BMI	Body mass index
DPP-4	Dipeptidyl peptidase-4
DPP-4i	Dipeptidyl peptidase-4 inhibitor
GLP-1	Glucagon-like peptide-1
GLP-1RA	Glucagon-like peptide-1 receptor agonist
HCC	Hepatocellular carcinoma
MASLD	Metabolic dysfunction-associated steatotic liver disease
MASH	Metabolic dysfunction-associated steatohepatitis
R0	Complete tumor resection with no residual tumor at the resection margin
T2D	Type 2 diabetes
